# Supplementary material for: A mental health paradox: Mental health was both a motivator and barrier to physical activity during the COVID-19 pandemic
Source: PLoS One. 2021 Apr 1;16(4):e0239244. doi: 10.1371/journal.pone.0239244 (PMC8016471; doi:10.1371/journal.pone.0239244)
Supplement: S1 Appendix — (DOCX) [file pone.0239244.s001.docx]

**S1 Appendix.**

**In the 6 months PRIOR to the COVID-19 crisis, what barriers did you face, if any, that prevented you from exercising? Please choose all that apply.**

I could not find the time in my day

I did not have access to a gym or recreational facility

I did not have the space or equipment necessary

I was too busy with child-care

I lacked the self-motivation to exercise

I lacked personal enjoyment of exercise

I lacked the financial flexibility for a gym membership

I experienced a recent injury

I had a fear of getting injured

I lacked encouragement, support, or companionship from family and/or friends

I lacked confidence in my ability to be physically active

I felt too anxious or stressed to engage in physical activity

Prior to COVID-19, I did not experience any barriers to exercise

No answer

Other

**In the 6 months PRIOR to the COVID-19 crisis, what motivations did you have, if any, to exercise? Please select all that apply.**

To maintain a healthy body weight

I had specific appearance goals My physician or a health-care specialist recommended it

To build muscle and/or strength

To increase my energy levels throughout the day

To reduce feelings of anxiety

To reduce stress

Social engagement (I.e group fitness classes, workout partners, trainers, etc)

To improve my sleep

For my personal enjoyment

For sport-specific training

Prior to COVID-19, I did not experience any motivation to exercise

No answer

Other

**SINCE the onset of the COVID-19 crisis, what barriers have you faced, if any, that have prevented you from exercising? Please choose all that apply.**

I cannot find the time in my day

I do not have access to a gym or recreational facilities

I do not have the space or equipment necessary

I am too busy with child-care

I lack the self-motivation to exercise

I lack personal enjoyment of exercise I lack the financial flexibility for a gym membership

I experienced a recent injury

I have a fear of getting injured

I lack encouragement, support, or companionship from family and/or friends

I lack confidence in my ability to be physically active

I feel too anxious or stressed to engage in physical activity

Since the COVID-19 crisis, I have not experienced any barriers to exercise

No answer

Other

**SINCE the onset of the COVID-19 crisis, what motivations have you had, if any, to exercise? Please select all that apply.**

To maintain a healthy body weight

I have specific appearance goals

My physician or a health-care specialist recommended it

To build muscle and/or strength

To increase my energy levels throughout the day

To reduce feelings of anxiety

To reduce stress

Social engagement (I.e group fitness classes, workout partners, trainers, etc)

To improve my sleep

For my personal enjoyment

For sport-specific training

Since the onset of COVID-19, I have not had any motivation to exercise

No answer

Other
